# Epirubicin sequential natural killer cells enhanced the cytotoxicity to breast cancer cells *in vitro*

**DOI:** 10.1186/2051-1426-3-S2-P6

**Published:** 2015-11-04

**Authors:** Yanqiu Song, Qian Wang, Hui Feng, Ying Dong, Jingtao Chen

**Affiliations:** 1 Cancer Center, The First Hospital, Jilin University, Changchun, 130061, Jilin, China; 2 Institute of Translational Medicine, The First Hospital, Jilin University, Changchun, 130061, Jilin, China

## Background

Anthracycline-based chemotherapy is a conventional therapy for breast cancer patients, but also negatively affects host immune function. When the host immune system does not work, chemotherapy and radiotherapy cannot kill the cancer cells efficiently. Therefore, improve the host immune system is important for cancer efficient treatment. Meanwhile, natural killer (NK) cells are well known to boost the immune responses against cancer.

## Methods

In this study, breast cancer cell lines were treated with anthracycline agent epirubicin (EPI) sequential with immune cells NK. NK cells in the study were amplified for 14 days *in vitro* from autologous adoptive cell transfer of breast cancer patients.

## Results

The cytotoxicity of NK cells against breast cancer cells with 12 hr EPI (5.0 μg/ml) pre-treatment was significantly higher than untreated or EPI alone. Thus, EPI sequential NK cells show the synergistic cytotoxicity effects against breast cancer cells. Moreover, breast cancer cells show increased expression of NKG2D ligands (ULBP1, ULBP2 and MICA) after treatment of EPI. Additionally, EPI sequential NK cells secret more IFN-γ and TNF-α as well as the increased expression of perforin and Granzyme B.

## Conclusions

Our findings reveal possible evidence for how to combine anthracycline-based chemotherapy with NK cells biotherapy to make a better treatment.

